# Purified Vitexin Compound 1 Serves as a Promising Antineoplastic Agent in Ovarian Cancer

**DOI:** 10.3389/fonc.2021.734708

**Published:** 2021-09-23

**Authors:** Kewen Ma, Kuansong Wang, Yingjun Zhou, Nian Liu, Wei Guo, Jialin Qi, Zhenmin Hu, Shitong Su, Ping Tang, Xunjian Zhou

**Affiliations:** ^1^ Department of Pathology, Xiangya Hospital, Central South University, Changsha, China; ^2^ Department of Pathology, School of Basic Medical Sciences, Central South University, Changsha, China; ^3^ School of Pharmaceutical Science, Central South University, Changsha, China; ^4^ Department of Dermatology, Xiangya Hospital, Central South University, Changsha, China; ^5^ Hunan Key Laboratory of Skin Cancer and Psoriasis, Xiangya Hospital, Central South University, Changsha, China

**Keywords:** vitexin compound 1, ovarian cancer, antineoplastic agent, therapy, apoptosis and cell cycle

## Abstract

Ovarian cancer is a common gynecologic aggressive neoplasm. The mortality of ovarian cancer is top among gynecologic malignancies due to the insidious onset, atypical early symptoms, and chemoresistance. Therefore, it is urgent to seek another promising treatment for ovarian cancer. Purified vitexin compound 1 (VB1) is a kind of neolignan from the seed of traditional Chinese herb vitex negundo that possessed diverse pharmacological effects. VB1 can exhibit anti-neoplastic activities against various cancers. However, the role of VB1 in ovarian cancer treatment has not been elaborated, and the mechanism is unknown. The aim of this study was to investigate the therapeutic effects of VB1 in ovarian cancer cells both *in vitro* and *in vivo*, along with the molecular mechanism of action. *In vitro*, VB-1 can effectively suppress the proliferation, induce apoptosis, and block cell cycle at G2/M phase with a concentration dependent manner in ovarian cancer cells. Western blot assay showed that VB1 induce apoptosis *via* upregulating expression of cleaved-caspase3 and block cell cycle at G2/M phase through upregulating expression of P21. Meanwhile, VB1 can effectively inhibit tumor growth in xenograft mouse model. Our research indicated that VB1 can significantly exert its anti-neoplastic effects and may represent a new class of agents in ovarian cancer therapy.

## Introduction

Ovarian cancer (OC) is a kind of highly heterogeneous disease that affects women globally, known as “silent killer”, and the overall mortality generally is higher than other gynecologic malignancies. It is estimated that 22,240 new cases will be diagnosed and 14,070 women will die of ovarian cancer in the United States in 2018 (1, 2). Most patients are being diagnosed in an advanced stage because ovarian cancer is often silent in its early stages (3). Ovarian cancer usually leads to peritoneal carcinoma (PC), which is another reason why the mortality of ovarian cancer is extremely high (4). Currently, cytoreductive surgery, chemotherapy, and targeted therapy have been applied to treat ovarian cancer and improve prognosis of patients with ovarian cancer to some extent (5, 6). Nevertheless, approximately 65% of patients with advanced-stage ovarian cancer relapse within 2 years after initial therapy (7). Ovarian cancer patients (15–25%) develop primary treatment resistance, and most of the remaining patients occur to chemotherapy resistance (8). Therefore, the development of more effective cancer therapy remains an ongoing challenge in ovarian cancer.

In China, traditional Chinese medicine is widely being used in the majority of hospitals and plays an active role in multiple diseases including cancers (9). Vitex negundo L is a powerful tonic traditional Chinese herb, possessing a wide range of biological activity against bacteria, tumor, rheumatism, etc. (10, 11). Vitexin compound 1 (VB1) is the most abundant Vitexin compound in EVn-50 which is derived from Vitex negundo L. It showed strong anti-neoplastic potential in cancers including colorectal cancer, hepatocellular carcinoma, and choriocarcinoma (12–16). Studies have shown that VB1 can suppress the tumor growth and angiogenesis by inactivating Akt signaling in hepatocellular carcinoma (17). VB1 can inhibit melanoma cell growth through DNA damage *via* increasing ROS levels (18).

Yet, for now, the effects and underlying mechanisms of VB1 on human ovarian cancers are largely unknown. The purpose of our study was to evaluate VB1 as anti-gynecological cancer herbs and to explore the molecular mechanism of anticancer activity. The result gained by our research may significantly contribute to the development of new and effective therapy for ovarian cancer.

## Materials and Methods

### Chemical

**Figure 1:**
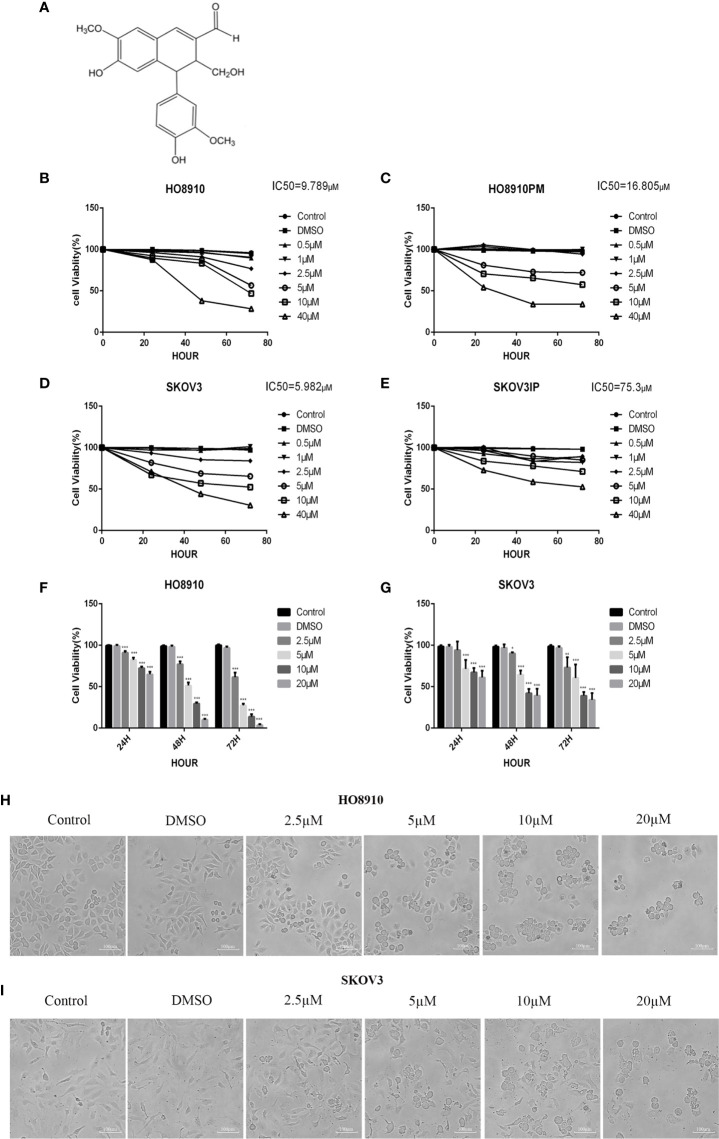
VB1 suppressed the viability of human ovarian cancer cells. **(A)** Chemical structure of VB1. **(B–E)** The four cell lines were treated with VB1 for various times and dosages, respectively, and the cell viability was tested by CCK-8. **(F)** HO8910 cells were treated with VB1 (0 µM) (control), 0.1% DMSO, and VB1 (2.5–20 µM) for 24, 48, and 72 h, and the cell viability was measured using CCK-8 assay. **(G)** SKOV3 cells were treated with VB1 (0 µM), 0.1% DMSO, and VB1 (2.5–20 µM) for 24, 48, and 72 h; the cell viability was measured using CCK-8 assay. **(H)** The cell morphologic changes of HO8910 cells after treatment with VB1 (0 µM), 0.1% DMSO, and VB1 (2.5–20 µM) for 24 h (magnification ×100). **(I)** The cell morphological changes after treatment with VB1 (0 µM), 0.1% DMSO, and VB1 (2.5–20 µM) for 24 h in SKOV3 cells (magnification ×100). The data represent the mean ± SD of three independent experiments, *P < 0.05, ***P < 0.001 compared with control.

VB1 termed 6-hydroxy-4-(4-hydroxy-3-methoxypheny1)-3-hydroxymethy1-7-methoxy-3,4-dihydro-2-naphthaldehyde, a purified compound, was extracted from the seed of the Chinese herb Vitex negundo and was supplied by the College of Pharmacy of Central South University (Changsha, China) (**Figure 1A**). VB1 was dissolved in dimethylsulfoxide (DMSO, Sigma) and diluted with pure water to a concentration of 10 mmol/L, which was packed and stored at 4°C. DMSO’s final concentration in each sample was less than 0.1% (vol/vol).

### Cell Lines and Culture Conditions

Human ovarian cancer cell lines HO8910 and SKOV3 were derived from American Type Culture Collection (ATCC). H08910 cell line was maintained in RPMI1640 medium (BI, Israel) supplemented with 10% fetal bovine serum (BI, Israel) at 37°C in a 5% carbon dioxide atmosphere. SKOV3 cell line was grown in McCoy’s5a medium (Gibco, USA) containing 10% fetal bovine serum (BI, Israel) at 37°C and 5% CO_2_.

### Cell Viability Assay

Cells were seeded into 96-well plates (5 × 10^3^ cells per well) and cultured at 37°C in media containing 10% FBS for 24 h. Cells were exposed to VB1 (0 µM), 0.1% DMSO, or various concentrations of VB1 (2.5, 5, 10, and 20 µM) for 24, 48, and 72 h, respectively. The cell viability (%) was determined by CCK-8 assay (7 sea biotech, China) with the manufacturer’s instructions. In detail, the reagent (10 μl) was added into each well of the 96-well assay plate containing the samples in 100 ml of culture medium and incubated for another 4 hours, after 24, 48, and 72 h individually. The absorbance at a wavelength of 450 nm was measured using a microplate absorbance reader (Eppendorf, GER). The half-maximal inhibitory concentration (IC50) values were generated using the SPSS software.

### Morphological Observation

Cells were seeded into six-well plates (3 × 10^5^ cells per well) and cultured at 37°C in media containing 10% FBS and 5% CO_2_ for 24 h. Subsequently, cells were subjected to VB1 (0 µM), 0.1% DMSO, or different concentrations of VB1 (2.5, 5, 10, and 20 µM) for 24 h. Cell morphological changes after treatment with VB1 were observed, and exact images were acquired using a microscope (Olympus, Japan).

### Hoechst 33258 Staining Assay

Cells were seeded into six-well plates (3 × 10^5^ cells per well) and cultured at 37°C in media containing 10% FBS for 24 h. Separately, cells were exposed to VB1 (0 µM), 0.1% DMSO, or various concentrations of VB1 (2.5, 5, 10, and 20 µM) for 24 h. After processing, cells were washed with PBS and fixed with 4% formaldehyde for 20 min. The fixed cells were stained with Hoechst 33258 for 10 min and washed with PBS. Finally, the apoptotic cells were observed and fluorescent micrographs were obtained using a fluorescent microscope (Olympus, Japan).

### Detection of Apoptotic Cells by Flow Cytometry

Cells were seeded into six-well plates (3 × 10^5^ cells per well) and incubated overnight at 37°C in media containing 10% FBS. Then, they were treated with VB1 (0 µM), 0.1% DMSO, or VB1 (2.5, 5, 10, and 20 µM) with the different concentrations for 24 h. The cells were harvested by trypsinization without EDTA after 24 h. The collected cells were suspended and washed with cold PBS, centrifuged, and stained with 5 µl AnnexinV and 5 µl propidium iodide for 15 min at room temperature in dark place according to Annexin V-FITC/PI Apoptosis Detection Kit (Becton, Dickinson and Company, USA) with the manufacturer’s instructions. The number of apoptotic cells was detected by flow cytometry and analyzed using the Flowjo software.

### Cell Cycle Assay

Cells were seeded into six-well plates (3 × 10^5^ cells per well) and incubated overnight at 37°C in media containing 10% FBS. Then, they were treated with VB1 (0 µM), 0.1% DMSO, or various of concentrations of VB1 (2.5, 5, 10, and 20 µM). The cells were harvested by trypsinization after 24 h. The collect cells were suspended and washed with cold PBS, centrifuged, and fixed in 70% ethanol at 4°C overnight. The next day, the fixed cells were suspended and centrifuged again; cells were incubated with 500 μl propidium iodide (PI) for 15 min at room temperature and protected from light according to the manufacturer’s instructions (Becton, Dickinson and Company, USA). The cell cycle was measured by flow cytometry and analyzed using ModFit software.

### Immunoblotting

SKOV3 cells were lysed in RIPA buffer (CWBIO, China) containing protease inhibitor cocktail (CWBIO, China) and phosphatase inhibitor cocktail (CWBIO, China) and incubated on ice for 20 min. Protein concentrations were determined using a BCA protein assay kit (DingGuo, China). Total protein extracted from SKOV3 cells were separated by 8–12% SDS-polyacrylamide gel electrophoresis (SDS-PAGE) and transferred to polyvinylidene fluoride membranes (Millipore, USA). After blocking in 5% non-fat dry milk in TBS at room temperature for 1 h, the membranes were incubated with primary antibodies including cleaved caspase3, caspase3, and P21 (Cell Signaling, Denver, MA) and β-actin (Proteintech, USA) overnight at 4°C. Subsequently, the membranes were washed with TBST and incubated with HRP-conjugated secondary antibody (Proteintech, USA) for 1 h at room temperature. Finally, the protein expression was imaged using a gel image analysis system (Bio-Rad, USA).

### Xenograft Tumor Model

The animal experiments were approved by the Animal Research Ethics Committee of Central South University. Twelve female BALB/c pathogen-free athymic nude mice at the age of 5 to 6 weeks were injected with a total of 5 × 10^6^ HO8910 cells subcutaneously into the right flank to establish a xenograft tumor model. When the tumors reached 5 mm^3^ or larger, the tumor-bearing mice were randomly divided into two groups and received vehicle or 80 mg/kg of VB1 by intraperitoneal injection twice a day for a month, with six mice in each group. Sizes of tumor were measured using a caliper every 3 days, and the tumor volume was calculated with the formula V = 1/2 (length × width^2^). Then the mice were sacrificed, and tumors were dissected and stored in liquid nitrogen or fixed in formalin for further histological analysis. All experiments were performed according to Guidelines for the Care and Use of Laboratory Animals prepared by the Institutional Animal Care and Use Committee of Central South University, Chang Sha, China. All Animal experimental protocols were approved by the Institutional Animal Care and Use Committee of Central South University (approval no. 2018sydw0225).

### Statistical Analysis

The data were expressed as the mean ± SD of three independent experiments. Statistical differences in the data were evaluated using a Student’s t-tests and ANOVA tests. P < 0.05 was considered as statistically significant.

## Results

### VB1 Suppressed the Viability of Human Ovarian Cancer Cells

In order to evaluate effect of VB1 on viability in ovarian cancer cell lines, we executed CCK-8 assay to measure cell viability after exposing them to 0.1% DMSO or increasing concentrations of VB1 for 24–72 h. HO8910 and SKOV3 have the most significant inhibitory effect, and their IC50 values of VB1 were 9.789 and 5.982 µM, respectively (**Figures 1B–E**). So we used HO8910 and SKOV3 as the test subjects. Results showed that VB1 possessed ability to suppress viability in ovarian cancer cell lines with a dose-dependent and time-dependent manner (**Figures 1F, G**). Compared with the VB1 (0 µM) group (Control) and 0.1% DMSO group, the cell viability of the VB1 group was significantly decreased. HO8910 cells were cultured with various concentrations of VB1 (2.5, 5, 10, and 20 µM) for 72 h, and the cell viability was 61.5%, 27.3%, 13.7%, and 3.5%, separately. In response to 20 μM of VB1 for 24, 48, and 72 h, the 20 μM VB1-treated H08910 cell viability was 64.8%, 9.9%, and 3.5%. SKOV3 cells were treated with VB1 (2.5–20 µM) for 72 h, and the cell survival rates were 73.1%, 60.5%, 39.1%, and 34.1%. SKOV3 cells in response to VB1 (20 µM) for 24, 48, and 72 h, the cell viability was 61.0%, 39.0%, and 34.1%. Meanwhile, VB1 treatment led to morphological changes in H08910 and SKOV3 cells (**Figures 1H, I**). Compared with the VB1 (0 µM) and 0.1% DMSO group, H08910 and SKOV3 cells were incubated with increasing concentrations of VB1 for 24 h, and the cell density decreased gradually. Adhered cells became small and round, and part of them were lysed after VB1 treatment. From the above data, we concluded that VB1 effectively suppressed the viability of human ovarian cancer cells, and VB1 may act as a promising anti-neoplastic agent in ovarian cancer.

### VB1 Provoked Apoptosis in Human Ovarian Cancer Cells With a Caspase Signaling Pathway

**Figure 2:**
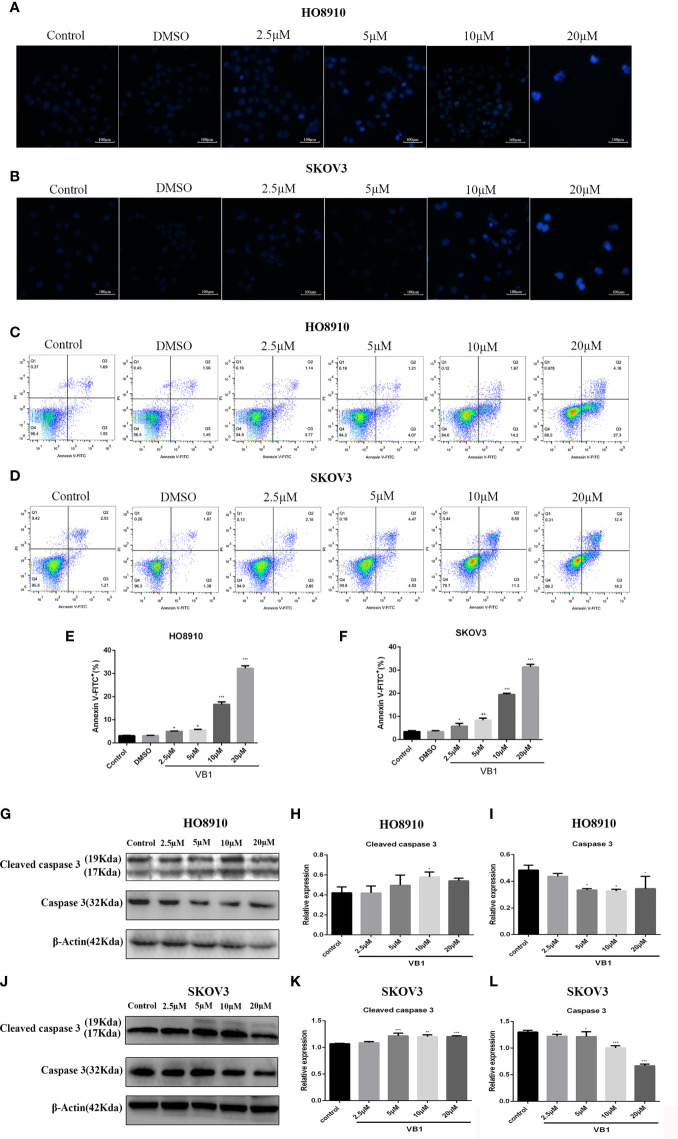
VB1 provoked apoptosis in human ovarian cancer cells with a caspase signaling pathway. **(A, B)** H08910 cells and SKOV3 cells were exposed to VB1 (0 µM), 0.1% DMSO, or VB1 (2.5–20 µM) for 24 h; after 24 h, the treated cells were subjected to Hoechst 33258 staining and apoptotic morphological changes were observed using a microscope (magnification ×100). **(C–F)** HO8910 cells and SKOV3 cells were handled with VB1 (0 µM), 0.1% DMSO, and VB1 (2.5–20 µM) for 24 h; the flow cytometry with Annexin V-FITC/PI staining was performed to detect apoptosis. **(G–I)** HO8910 cells were exposed to VB1 (0-20 µM) for 24 h; the expression of apoptotic protein was determined by Western blotting. **(J–L)** SKOV3 cells were exposed to VB1 (0–20 µM) for 24 h, and the expression of apoptotic protein was determined by Western blotting. Data represent the mean ± SD of three independent experiments. *P < 0.05, **P < 0.01, ***P < 0.001 compared with the control group.

Our preliminary data showed that VB1 could effectively suppress the viability of human ovarian cancer cells in a concentration way, so we speculate that whether VB1 inhibit the viability by provoking apoptosis? Firstly, we observed the apoptotic morphological changes of HO8910 and SKOV3 cells that were exposed to VB1 (0 µM), 0.1% DMSO, and VB1 (2.5–20 µM) for 24 h using a microscope. Typical apoptotic features were observed by us, such as nuclear debris, aberrant nuclear shape, and nuclear division. Apoptotic cells show strong blue staining, and exact images were observed in **Figures 2A, B**. Subsequently, after treatment with VB1 (0 µM), 0.1% DMSO, or VB1 (2.5–20 µM) for 24 h in both HO8910 and SKOV3 cells, apoptosis was detected by the flow cytometry. Compared with the group of VB1 (0 µM) (control) and 0.1% DMSO, the number of apoptotic cells significantly increased in a dose-dependent manner (**Figures 2C–F**). After processing with VB1 (2.5–20 µM) for 24 h, the apoptosis rate of HO8910 cells was 4.91%, 5.28%, 15.87%, and 31.46%, respectively. The percentage of apoptotic cells in SKOV3 cells were 5%, 9%, 19.85%, and 30.6%, separately. Furthermore, in order to determine the molecular mechanisms of VB1-induced apoptosis in HO8910 cells and SKOV3 cells, we explored the expression of apoptosis-related proteins including caspase-3 and cleaved caspase-3 by Western blotting. After treatment with VB1 (0–20 µM) for 24 h, groups of various concentrations of VB1 showed caspase-3 decreased and cleaved caspase-3 increased in HO8910 cells (**Figures 2G–I**). Similarly, VB1 (0–20 µM) treatment for 24 h decreased caspase-3 and increased cleaved caspase-3 in SKOV3 cells with a concentration-dependent manner, compared with the VB1 (0 µM) group (**Figures 2J–L**). In conclusion, our findings were enough to prove VB1 exactly induced apoptosis with a caspase signaling pathway.

### VB1 Induces G2/M Arrest in Human Ovarian Cancer Cells

**Figure 3:**
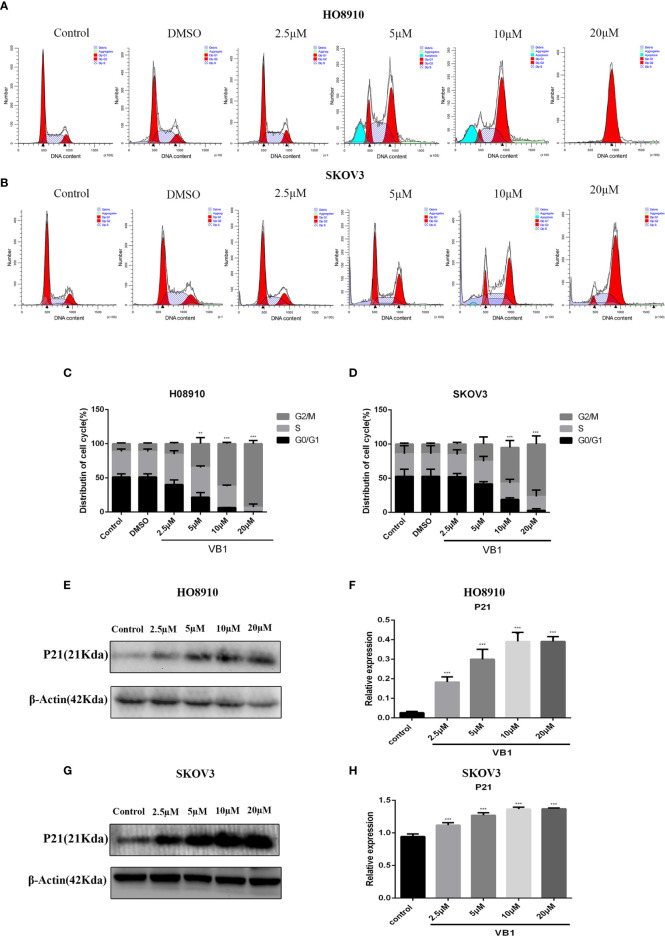
VB1 induces G2/M arrest in human ovarian cancer cells. **(A–D)** H08910 and SKOV3 cells were exposed to VB1 (0 µM), 0.1% DMSO, and VB1 (2.5–20 µM) for 24 h; the flow cytometry was performed to detected distribution of cell cycle. **(E, F)** The expression of P21 in HO8910 cells which were treated with VB1 (0–20 µM) for 24 h. Western blotting was performed by p21 antibody. **(G, H)** The expression of P21 in SKOV3 cells which were treated with VB1 (0–20 µM) for 24 h, Western blotting was performed by p21 antibody. The results represent the mean ± SD of three independent experiments. **P < 0.01, ***P < 0.001 compared with the control group.

To delineate the effects of VB1 on cell cycle in HO8910 and SKOV3 cells, cell cycle assay was conducted by the flow cytometry. Compared with the group of VB1 (0 µM) and 0.1% DMSO, we found that VB1 could dramatically induce G2/M arrest in a concentration-dependent way (**Figures 3A–D**). HO8910 cells were treated with VB1 (2.5–20 µM) for 24 h, and the percentage of cells arrested in G2/M phase were 14.21%, 41.14%, 60.94%, and 96.38%, respectively. Parallelly, the arrest rant of SKOV3 cells was 17.19%, 32.75%, 44.99%, and 68.17%, individually. Our data suggested that VB1 markedly blocked cell cycle at G2/M phase by decreasing the distribution of G0/G1 phase. To the best of our knowledge, cyclin B and CDK1 play a significant role in regulation of progression of G2/M phase. P21, a downstream molecule of P53, could inhibit activity of cyclin-dependent kinases and arrest cell cycle at G2/M phase by connecting with cyclinB-CDK1 complex. Therefore, we next examined the expression of P21 by Western blotting. The results showed that the expression of P21 was elevated after treatment with the increasing concentrations of VB1 for 24 h in SKOV3 and HO8910 cells (**Figures 3E–H**). These data indicated that VB1 induces G2/M arrest *via* upregulation of the expression of p21.

### VB1 Can Inhibit Tumor Growth in a Xenograft Tumor Model

**Figure 4:**
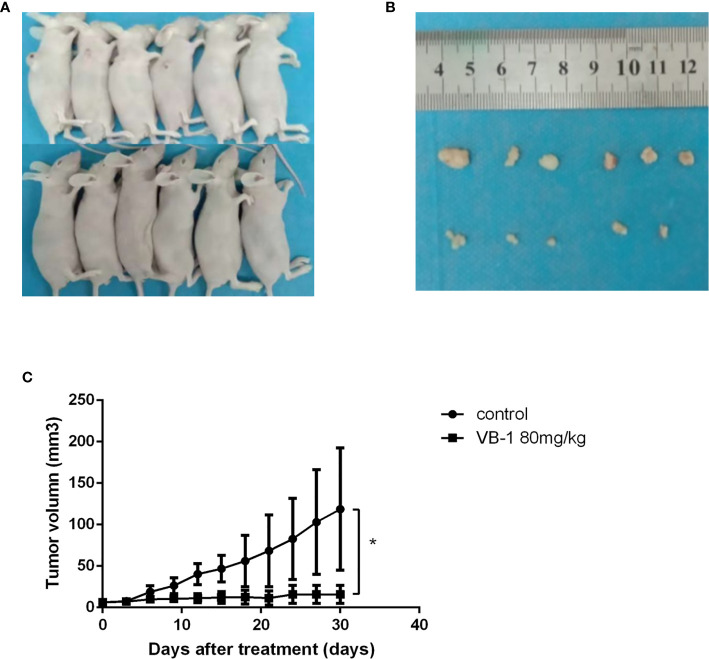
VB1 can inhibit tumor growth in a xenograft tumor model. **(A)** Tumor-bearing nude mouse model after treatment. **(B)** VB1-treated mice demonstrated a dramatically reduced tumor volume as compared with vehicle-treated mice. **(C)** Tumor growth curves indicate a poor tumor growth in VB1-treated mice compared with vehicle-treated mice. The results represent the mean ± SD per group. *P < 0.05 compared with the control group.

Our data showed that VB-1 could effectively suppress the proliferation of HO8910 and SKOV3 cells, as well as induce apoptosis and block cell cycle at G2/M phase *in vitro*. Hence, we guessed that VB1 whether could inhibit tumor growth *in vivo*? We assessed VB1’s antitumor effects *in vivo* by an animal model study. Similarly, VB1 could also significantly inhibit tumor growth, compared with the vehicle-treated group (**Figures 4A, B**). The tumor size of vehicle-treated group increased gradually during treatment, and the average tumor size was 118.7mm^3^ after treatment. Tumor size of 80 mg/kg doses of VB1-treated group has no significant change, and the average tumor size was 19.0mm^3^ which was smaller than the vehicle-treated group (**Figure 4C**). Meanwhile, we found a tumor of tumor-bearing mice disappeared in VB1-treated group. Consistent with the results *in vitro*, the data from experiment *in vivo* indicated the inhibitory effect of VB1 on tumor growth.

## Discussion

In our study, we found that VB1 extracted from traditional Chinese herb vitex negundo possesses powerful anti-neoplastic effects on human ovarian cancer. Cell viability assay showed that VB1 could effectively suppress the proliferation of ovarian cancer cell lines in a time-dependent and dose-dependent manner. Flow cytometry analysis indicated that VB1 suppress the proliferation of human ovarian cancer cells by provoking apoptosis and blocking cell cycle at G2/M phase. Hoechst 33258 staining assay suggested that typical apoptotic features emerged after VB1 treatment in HO8910 and SKOV3 cells. Furthermore, Western blot analysis revealed underlying molecular mechanisms of anti-tumor effects of VB1 on HO8910 and SKOV3 cells. VB-1 exerted its anti-neoplastic effects through upregulating the expression of cleaved-caspase3 and p21. Not only could VB1 possess inhibitory activities *in vitro*, but also inhibit tumor growth in a ovarian cancer subcutaneous xenograft tumor model of nude mice.

Apoptosis, the process of programmed cell death, is usually characterized by specific morphological and distinct biochemical and genetic pathways (19). Apoptosis play a vital role in healthy balance between cell survival and cell death (20, 21). The classic channels of provoking apoptosis by caspase cascade include endogenous mitochondrial signaling pathway and exogenous death receptor signaling pathway. Once activation of cellular stress signals in mitochondrial signaling pathway, outer mitochondrial membrane integrity will be destroyed, and cytochrome c will be released from mitochondria to cytoplasm. Then, oligomerized adaptor protein Apaf-1 recruits initial caspase (caspase 9) to active downstream executioner caspases (caspase 3) and triggers apoptosis by cleaving poly ADP-ribose polymerase (22, 23).

The exogenous pathway, a receptor-mediated pathway, and these related death receptors are members of the tumor necrosis factor (TNF) receptor gene superfamily (24). The death-inducing signaling complex (DISC) is composed of receptor, corresponding ligand, adapter protein, and procaspase-8. When exogenous pathway was activated by death signal, the receptors will bind to homologous ligand, and the connection ligand and receptor results in recruitment of adapter protein, adapter protein further connecting procaspase-8. Subsequently, the DISC activated executioner caspases by cleaved target proteins and further led to the cell death (25). In our study, Western blot analysis showed that VB1 induced the elevated expression of cleaved caspase 3 in SKOV3 cell line and HO8910 cell line after VB1 treatment, which suggested that VB1 induce apoptosis of ovarian cancer with a caspase signaling pathway by participating endogenous mitochondrial and exogenous death receptor signaling pathway.

As we have seen, cell cycle is modulated by various cyclin/cyclin-dependent kinases complexes in eukaryotes, and each cyclin/cdk complex exerts specific effects on individual cell cycle phase. Cyclin B and CDK1 play a crucial role in regulating G2/M phase process. CyclinB–CDK1 complex was recognized as a M phase-promoting factor (MPF); it is capable of starting the G2/M phase transition and promotion of mitotic entry (26, 27). P53, a tumor suppressor and transcription factor, is involved in progresses and developments of malignancy cells. In p21Waf1/Cip1, a downstream molecule of P53, the connection between p21Waf1/Cip1 and cyclinB–CDK1 complex inhibits cyclin-dependent kinases and arrests cell cycle at G2/M phase (28). Western blot analysis showed that VB1 markedly increased the level of P21 with a dose-dependent manner, which implied that P53 may participate in the process of cell cycle after VB1 treatment in ovarian cancer.

Our study proved that VB1 could effectively suppress activities of ovarian cancer *in vitro* and *in vivo*; however, cellular toxicity of VB1 in human ovarian epithelial cells is unclear. If VB1 show little influence on normal ovarian epithelial cells, intolerable toxicity or side effects of VB1 are slighter than chemotherapeutic drugs.

In summary, our present study demonstrated that VB1 exerted anti-neoplastic activities *in vitro* by inhibiting proliferation, inducing apoptosis, and arresting cell cycle at G2/M phase. Meanwhile, VB1 suppressed tumor growth in a subcutaneous xenograft tumor model of nude mice. Further investigation showed that VB-1 may be *via* upregulating expression of cleaved-caspase3 and P21 to induce apoptosis and block cell cycle at G2/M phase. The data from our experiments suggested that VB1 was qualified to be a promising candidate for the therapy of human ovarian cancer in the future.

## Data Availability Statement

The original contributions presented in the study are included in the article/supplementary material. Further inquiries can be directed to the corresponding author.

## Ethics Statement

The animal study was reviewed and approved by Animal Care and Use committee of Central South University.

## Author Contributions

KM and NL were responsible for the design of the study, KM, JQ, and ZH performed the experiments. KW contributed to the review and revision of the manuscript. YZ extracted and purified drugs. KM, JQ, and PT wrote the first draft of the manuscript. WG, SS, and XZ analyzed the data. All authors discussed the results and commented on the manuscript. All authors contributed to the article and approved the submitted version.

## Funding

This work was supported by the Natural Science Foundation of China (Grant No. 81972490).

## Conflict of Interest

The authors declare that the research was conducted in the absence of any commercial or financial relationships that could be construed as a potential conflict of interest.

## Publisher’s Note

All claims expressed in this article are solely those of the authors and do not necessarily represent those of their affiliated organizations, or those of the publisher, the editors and the reviewers. Any product that may be evaluated in this article, or claim that may be made by its manufacturer, is not guaranteed or endorsed by the publisher.
